# Longitudinal Monitoring of Brain Volume Changes After COVID-19 Infection Using Artificial Intelligence-Based MRI Volumetry

**DOI:** 10.3390/diagnostics15243244

**Published:** 2025-12-18

**Authors:** Zeynep Bendella, Catherine Nichols Widmann, Christine Kindler, Robert Haase, Malte Sauer, Michael T. Heneka, Alexander Radbruch, Frederic Carsten Schmeel

**Affiliations:** 1 Department of Neuroradiology, Faculty of Medicine, University Hospital Bonn, Rheinische Friedrich-Wilhelm-Universität Bonn, 53127 Bonn, Germany; robert.haase@ukbonn.de (R.H.); maltesauer@gmx.net (M.S.); alexander.radbruch@ukbonn.de (A.R.);; 2 German Centre for Neurodegenerative Diseases (DZNE), 53127 Bonn, Germany; catherine.widmann@ukbonn.de (C.N.W.); michael.heneka@ukbonn.de (M.T.H.); 3 Department of Neurodegenerative Diseases and Geriatric Psychiatry, University Hospital Bonn, Rheinische Friedrich-Wilhelms-Universität Bonn, Venusberg-Campus 1, 53127 Bonn, Germany; 4 Department of Parkinson, Sleep and Movement Disorders, University Hospital Bonn, Rheinische Friedrich-Wilhelms-Universität Bonn, Venusberg-Campus 1, 53127 Bonn, Germany; christine.kindler@email.de; 5 Department of Vascular Neurology, University Hospital Bonn, Rheinische Friedrich-Wilhelms-Universität Bonn, Venusberg-Campus 1, 53127 Bonn, Germany; 6 Luxembourg Centre for Systems Biomedicine, L-4365 Esch-sur-Alzette, Luxembourg

**Keywords:** SARS-CoV-2, COVID-19, magnetic resonance imaging, brain atrophy, artificial intelligence, hippocampal volume

## Abstract

**Background/Objectives**: SARS-CoV-2 infection has been linked to long-term neurological sequelae and structural brain alterations. Previous analyses, including baseline results from the COVIMMUNE-Clin study, showed brain volume reductions in COVID-19 patients. Longitudinal data on progression are scarce. This study examined brain volume changes 12 months after baseline MRI in individuals who have recovered from mild or severe COVID-19 compared with controls. **Methods**: In this IRB-approved cohort study, 112 out of 172 recruited age- and sex-matched participants (38 controls, 36 mild/asymptomatic 38 severe COVID-19) underwent standardized brain MRI 12 months after baseline. Volumetric analysis was performed using AI-based software (mdbrain). Regional volumes were compared between groups with respect to absolute and normalized values. Multivariate regression controlled for demographics. **Results**: After 12 months, a significant decline in right hippocampal volume was observed across all groups, most pronounced in severe COVID-19 (SEV: Δ = −0.32 mL, *p* = 0.001). Normalized to intracranial volume, the reduction remained significant (SEV: Δ = −0.0003, *p* = 0.001; ASY: Δ = −0.0001, *p* = 0.001; CTL: minimal reduction, Δ ≈ 0, *p* = 0.005). Minor reductions in frontal and parietal lobes (e.g., right frontal SEV: Δ = −1.35 mL, *p* = 0.001), largely fell within physiological norms. These mild regional changes are consistent with expected ageing-related variability and do not suggest pathological progression. No widespread progressive atrophy was detected. **Conclusions**: This study demonstrates delayed, severity-dependent right hippocampal atrophy in recovered COVID-19 patients, suggesting long-term vulnerability of this memory-related region. In contrast, no progression of atrophy in other areas was observed. These findings highlight the need for extended post-COVID neurological monitoring, particularly of hippocampal integrity and its cognitive relevance.

## 1. Introduction

The severe acute respiratory syndrome coronavirus 2 (SARS-CoV-2), which causes COVID-19 [1,2], has caused an extensive number of infections globally [3]. A considerable proportion of affected individuals develop persistent symptoms, collectively termed post-COVID conditions or long COVID [4,5,6], which may occur irrespective of initial disease severity [7,8] and involve multiple organ systems [9]. Neurological manifestations constitute one of the most prevalent components of this syndrome, including cognitive, affective, and sensorimotor disturbances [10,11,12,13].

Compared with our previously published baseline analysis [14] and other early post-COVID imaging studies [15,16], the present work provides novel longitudinal evidence by examining whether structural brain changes progress, stabilize, or reverse over a full 12-month interval.

Several cohorts have reported persistent sensorimotor abnormalities, such as impaired fine motor control, slowed movements, balance difficulties, and reduced proprioception, in some cases partially resembling extrapyramidal features [10,11], suggesting dysfunction of cortico-subcortical motor networks. Neuroimaging studies have demonstrated microstructural white matter alterations [12], cortical thinning, and regional hypometabolism in frontal, parietal, and temporal lobes [13], providing structural correlates for these deficits. These findings are consistent with our previously published baseline analysis of the COVIMMUNE cohort, in which we observed widespread supratentorial grey matter reductions following COVID-19 infection [14].

Despite growing evidence of neurocognitive and sensorimotor sequelae after SARS-CoV-2 infection [10,11,12,13,14], the long-term structural brain trajectory of COVID-19 survivors remains insufficiently understood. Most available studies have been cross-sectional or have investigated early post-acute stages [14,15,16,17,18,19,20,21], leaving it unclear whether COVID-19-associated alterations stabilize, normalize, or progress over longer intervals. Moreover, previous work, including our baseline analysis, has not addressed whether specific regions, such as the hippocampus, follow distinct temporal patterns compared with global or lobar volumes. Clarifying these trajectories is clinically relevant, as hippocampal integrity is central to memory and attentional functions, which are among the most frequently reported complaints in long-COVID [22,23]. Therefore, the present study aimed to quantify 12-month longitudinal volumetric changes using automated AI-based imaging biomarkers in a clinically stratified cohort of COVID-19 survivors and matched controls.

A large-scale study conducted in England involving 606,434 participants revealed that a significant number of COVID-19 survivors reported experiencing one or more of 29 identified symptoms, with many reporting three or more up to 12 weeks after their initial illness [24]. Understanding the long-term consequences of COVID-19 on brain structure requires not only early post-recovery assessments but also continued longitudinal monitoring to capture possible ongoing neurological alterations. While initial studies have provided valuable insights into abnormalities in brain volume among patients recovering from COVID-19 [14,15,16], there is limited data on how these changes may progress over time. Recent findings suggest that COVID-19 may lead to sustained alterations in brain structure, contributing to persistent neurological, cognitive, and psychiatric symptoms in many patients [17,18]. To date, a number of systematic reviews have highlighted abnormalities on brain imaging as a major feature of COVID-19 [19,20,21].

This study constitutes a prospective study within the COVIMMUNE Umbrella project. The participants were re-examined 12 months after baseline MRI, which was published recently [14]. Our baseline MRI analysis demonstrated significant brain volume reduction following COVID-19 infection, aligning with observations reported in multiple independent cohorts [14,15]. In order to investigate potential long-term effects, this follow-up study assesses brain volume changes one year after the baseline MRI in the same cohort of patients. By comparing volumetric MRI data from patients with mild and severe cases of COVID-19 to healthy controls, we aimed to understand whether these brain abnormalities might potentially progress, improve, or stabilize over time. Therefore, the main question that we addressed was to determine whether subsequent assessments indicate ongoing structural decline, which could hint at longer-term neurodegenerative mechanisms. This longitudinal approach may shed light on the trajectory of COVID-19-related brain changes and help identify potential factors that contribute to ongoing neurological sequelae.

## 2. Materials and Methods

This single-centre study at University Hospital Bonn was funded by the German Ministry of Health. It comprised three subprojects. The present work reports the radiology-focused subproject. In addition to clinical and neuropsychological assessments conducted by trained medical professionals at three intervals (baseline, 6 months, and 12 months), participants underwent standardized brain MRI scans at baseline and 12 months post-enrollment.

The study protocol was reviewed and approved by the Medical Ethics Review Board of the University Hospital Bonn (approval ID: 511/20) on 10 March 2021. All participants provided written informed consent before study procedures commenced. The study was preregistered in the German Clinical Trials Registry (DRKS00023806) on 16 March 2021, and listed with the World Health Organization’s International Clinical Trials Registry Platform (ICTRP).

### 2.1. Study Population

At baseline, a total of 172 participants were prospectively recruited with similar age and sex distributions using frequency matching, by 10 March 2022, of whom 10 failed screening and 7 were lost during the scheduled MRI examination stage due to claustrophobia (*n* = 4) or metallic implants (*n* = 3). Due to non-attendance at the scheduled follow-up MRI, the final study sample currently consisted of 112 individuals who underwent the follow-up MRI 12 months after initial study enrollment. This final study sample was divided into three groups: 38 healthy control subjects, 36 participants with asymptomatic or mild COVID-19, and 38 participants who had experienced a severe course of the disease, as seen in Table 1.

To minimize the likelihood of including individuals with an undetected asymptomatic SARS-CoV-2 infection, all healthy controls underwent a SARS-CoV-2 rapid antibody test at screening, as specified in the study protocol. Only individuals without serological evidence of prior natural infection were included, and vaccination-related antibody positivity did not constitute an exclusion criterion. COVID-19 vaccination status, including vaccine type and timing, was not systematically recorded and was therefore not included as a covariate in the present analyses.

The entire study protocol has been described in detail previously [16], with the radiology project arm summarized as follows:

General exclusion criteria:

• Contraindications for MRI;
• Severe or unstable medical conditions;
• Current major depressive episode;
• Diagnosis of psychotic disorders, bipolar disorder, or history of substance abuse;
• Known neurodegenerative diseases (e.g., Alzheimer’s, Parkinson’s, frontotemporal dementia, Huntington’s disease, amyotrophic lateral sclerosis);
• Vascular dementia or history of stroke;
• History of malignant disease.

### 2.2. Magnetic Resonance Imaging

Each participant underwent a standardized brain MRI scan at study enrollment, which corresponded to the first imaging time point. For COVID-19-recovered, this scan took place after recovery, at a variable interval following infection (average 7–9 months) and 12 months after baseline MRI; control participants had no history of COVID-19. Imaging was performed on a clinical-grade 3 Tesla MRI scanner (Achieva TX, Philips Healthcare, Best, The Netherlands), using an 8-channel head coil. The same scanning protocol was applied for all participants. Sequences included three-dimensional (3D) T1-weighted magnetization-prepared rapid acquisition gradient echo (3D MPRAGE), 3D fluid-attenuated inversion recovery (3D-FLAIR), diffusion-weighted imaging (DWI), susceptibility-weighted imaging (SWI), and T2-weighted imaging (T2W). Detailed MRI acquisition parameters are provided in Table 2.

Although volumetric analyses were exclusively based on the 3D T1-weighted MPRAGE sequence, all additional sequences (T2W, FLAIR, DWI, and SWI) were systematically evaluated by board-certified neuroradiologists to screen for structural abnormalities that could confound volumetric interpretations, such as acute ischemia, microbleeds, or inflammatory lesions.

### 2.3. Image Analysis

Board-certified neuroradiologists reviewed all participants’ MRI scans to check for acute brain abnormalities and determine study eligibility.

### 2.4. Post-Processing and Artificial Intelligence (AI)-Based Volumetry

In this study, we used the CE-certified mdbrain software v.4.4.1, which relies on an internally developed deep-learning framework for automated brain segmentation. Its core component is a three-dimensional U-Net-based convolutional network that processes standardized 3D T1-weighted MRI data. Before being fed into the model, the scans undergo pre-processing steps that include cropping to the intracranial region and resampling to a uniform spatial resolution. The network then generates a volumetric label map covering all anatomical structures listed in Table 3 and Table 4.

The algorithm underlying the segmentation engine was trained on 2869 MRI datasets that had been manually annotated by several expert raters within a controlled multi-rater workflow. During optimization, the developers used the Adam algorithm and applied a diverse set of image augmentations, such as modifications in contrast, resolution, rotational orientation, and elastic deformation, to improve robustness and generalisability across scanners and acquisition settings. The final network configuration was evaluated on an independent test set of 121 scans, each with corresponding expert-derived ground truth labels.

After the segmentation step, the label map is converted into volumetric measures by counting the number of voxels associated with each region and multiplying the resulting voxel counts by the native voxel volume.

These volumes are then compared with values in mdbrain’s normative reference dataset, which includes 6099 healthy individuals (balanced for sex, age range 10–97 years, mean age 41 ± 23 years) recruited from multiple imaging centres across Europe, North America, Australia, and China. Percentile values are derived by adjusting for age, sex, and total intracranial volume, enabling an individualized assessment relative to the population baseline.

According to the vendor documentation, reliable performance of the software requires that MRI acquisition parameters fall within specific technical limits (e.g., slice spacing < 2 mm) and that the algorithm is used within its validated age range (10–99 years). Accuracy may be compromised in the presence of major intracranial pathology (such as neoplasms or large territorial strokes) or when baseline and follow-up scans originate from substantially different scanner models or acquisition protocols.

In the present study, all AI-generated segmentations were independently reviewed by two board-certified neuroradiologists to verify anatomical correctness and to exclude potential segmentation artefacts. Any inconsistencies identified during this quality control step were jointly resolved through consensus.

### 2.5. Statistical Analysis

All statistical evaluations were performed using SPSS (Version 27 or higher; IBM Corp., Armonk, NY, USA) and the R statistical environment (v4.2.2, R core Team, R Foundation for Statistical Computing, Vienna, Austria, URL: https://www.R-project.org/, accessed on 15 March 2023). In R, the jtools package (v2.2.0, URL: https://cran.r-project.org/package=jtools, accessed on 15 March 2023) was used for generating regression summaries and effect displays.

To examine distributional properties of the volumetric variables, we applied the Shapiro–Wilk test, which indicated that several measurements deviated from normality. As a consequence, non-parametric Kruskal–Wallis tests were used for group comparisons. Unless noted otherwise, demographic and imaging parameters are reported as mean ± standard deviation. Given the large number of regional measurements, we adopted a conservative significance criterion of *p* < 0.005. Findings with *p*-values between 0.005 and 0.05 were documented as descriptive trends uncorrected results but were not interpreted as statistically significant, reflecting the exploratory character of the analyses. No formal Bonferroni correction was applied given the exploratory nature of the analyses.

A priori statistical power analyses were performed and recently described elsewhere [16], yielding a required total sample size of 126 at an estimated actual power of 80%. A priori sample-size estimation was performed using G*Power 3.1 for repeated-measures ANOVA with within-between interaction (effect size f = 0.20, α = 0.05, power = 0.80), resulting in a required total sample of *N* = 126 [16].

We assessed longitudinal differences in brain volume using a repeated-measures ANOVA with time (baseline vs. 12 months) as the repeated factor and group (CTL, ASY, SEV) as the grouping variable. Individual changes between the two time points were quantified by calculating the absolute difference delta (Δ) between 12-month follow-up and baseline measurements (i.e., Δ = volume follow-up-volume baseline).

To further analyze which variables contributed to the observed volumetric differences, we constructed multivariate regression models. These models incorporated age, sex, height, body mass index (BMI), and COVID-19 severity indicators (ASY for asymptomatic/mild courses and SEV for severe cases) as predictors. For each of these predictors, the regression produced a numerical estimate that describes both the strength and the direction of its association with the respective brain volume measure. The estimate represents the expected change in regional volume (in mL) associated with a one-unit increase in the predictor, such as one additional year of age, one centimetre of height, one kg/m^2^ of BMI, or one categorical step on the ASY/SEV severity scale, while holding all remaining predictors constant.

For each model, we report the regression coefficient, its standard error, the corresponding t-value and *p*-value, as well as the R^2^ statistic, which indicates how much of the variance in regional brain volume can be explained by the combined predictors.

## 3. Results

The final study cohort comprised 112 participants successfully completed the 12-month follow-up MRI and were distributed across three subgroups: healthy controls (CTL, *n* = 38), individuals with asymptomatic or mild COVID-19 (ASY, *n* = 36), and patients with severe disease requiring hospitalization (SEV, *n* = 38). The duration between infection and study participation at baseline ranged from 8.7 ± 4.8 months for the ASY group to 10.7 ± 5 months for the SEV group at the time of evaluation, and the second MRI was performed 12 months after the first MRI.

Table 1 provides an overview of the demographic and clinical characteristics of the cohort. No significant differences were observed among the three sub-cohorts with respect to age, gender distribution, weight, height, or BMI. Nonetheless, participants in the SEV group tended to be older on average compared with those in the CTL and ASY groups. Most individuals in the SEV group had been treated in standard wards or monitoring units, with 9 out of 38 participants (23.7%) requiring admission to an intensive care unit (ICU).

### Volumetric Brain Analysis

Volumetric outcomes are reported in the predefined regional hierarchy: hippocampal volumes first, followed by lobar compartments and subsequently other cortical and subcortical regions.

As in the baseline study, no abnormalities were detected during the visual clinical assessment that warranted the exclusion of any subjects based on the defined exclusion criteria. The volumetry software successfully analyzed all MRI studies, providing measurements of brain region and ventricle volumes in millilitres (mL) together with their corresponding percentiles for the three participant groups in the follow-up assessments conducted 12 months after the first MRI: CTL, ASY, and SEV (Table 3 and Table 4). In contrast to the baseline MRI [14], which showed widespread significant brain volume reductions particularly in the supratentorial grey matter, both frontal and parietal lobes, and the right thalamus, the current longitudinal follow-up identified a more localized and temporally dynamic pattern of volumetric changes.

At baseline, SEV appeared to show slightly higher relative right-hippocampal values compared with CTL and ASY. These small differences are most likely explained by sampling factors (e.g., age distribution, comorbidities, head size), normal segmentation variability, or chance effects from multiple ROI comparisons, rather than by genuine structural enlargement.

In the 12-month follow-up assessments, significant differences were observed, most notably in the right hippocampus across all three groups. This reduction was most pronounced in the SEV group and showed a clear severity-dependent gradient. In absolute terms, mean right hippocampal volume decreased in CTL from 4.21 ± 0.45 mL to 4.04 ± 0.38 mL (Δ = −0.17 mL, *p* = 0.001), in ASY from 4.30 ± 0.47 mL to 4.06 ± 0.37 mL (Δ = −0.24 mL, *p* = 0.001), and in SEV from 4.21 ± 0.44 mL to 3.89 ± 0.31 mL (Δ = −0.32 mL, *p* = 0.001). Normalized to ICV, corresponding decreases were CTL: Δ = 0.00004 (*p* = 0.005), ASY: Δ = −0.0001 (*p* = 0.001), SEV: Δ = −0.0003 (*p* = 0.001).

**Figure 1:**
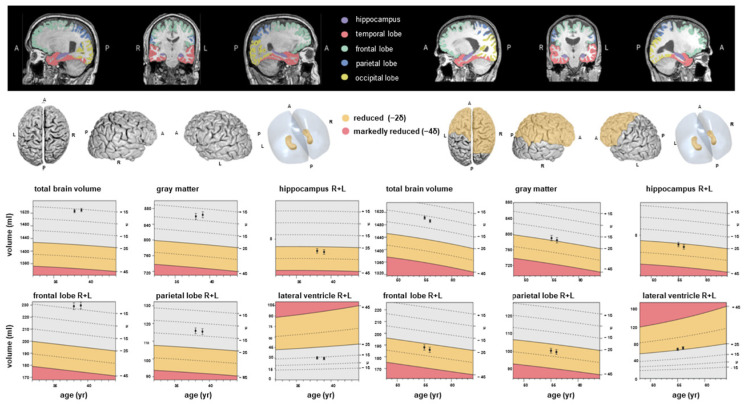
Examples of fully automated, artificial intelligence (AI)-based brain volumetry in two patients with severe course of COVID-19 (left: patient 1, right: patient 2) in time sequelae: baseline and follow-up MRI. Various brain volumes are displayed together with their deviations from age- and sex-adjusted normative values. The colour scale indicates the degree of volume reduction, with yellow representing moderate deviation (~2 SD) and red indicating marked deviation (~4 SD). A, anterior, P, posterior, L, left, R, right.

This hippocampal atrophy was not observed in the baseline MRI, highlighting a delayed and potentially progressive effect of SARS-CoV-2 infection on hippocampal integrity. Representative volumetric MRI findings are illustrated in Figure 1.

**Frontal lobes:** In absolute terms, right frontal lobe volume decreased in CTL from 91.38 mL to 89.50 mL (Δ = −1.88 mL, *p* = 0.001), in ASY from 91.24 mL to 89.89 mL (Δ = −1.35 mL, *p* = 0.001), and in SEV from 84.63 mL to 83.28 mL (Δ = −1.35 mL, *p* = 0.001). For the left frontal lobe, the decrease was more modest (SEV: 81.44 mL to 80.64 mL, Δ = −0.80 mL, *p* = 0.004). However, ICV-normalized differences were smaller and less consistently significant, particularly in SEV (Δ = −0.0006, *p* = 0.064). These results suggest stable or only mildly progressive changes in frontal regions over time compared with the baseline MRI, which showed early and significant group differences in these areas. The colour scale reflects the AI-derived deviation of regional brain volumes from age- and sex-adjusted normative values, with warmer colours indicating relative volume decrease and cooler colours indicating relative preservation.

**Temporal lobes:** Right temporal lobe volumes declined modestly (CTL: Δ = −0.71 mL, *p* = 0.001; ASY: Δ = −0.96 mL, *p* = 0.001; SEV: Δ = −0.89 mL, *p* = 0.001), whereas ICV-normalized differences did not reach statistical significance. Changes in the left temporal lobe were less pronounced and likewise non-significant in normalized analysis. Unlike the baseline MRI, which revealed significant cross-sectional differences in both temporal lobes, the longitudinal follow-up indicates largely stable temporal lobe volumes.

**Parietal lobes:** The right parietal lobe volume declined from 48.11 mL to 47.25 mL in CTL (Δ = −0.86 mL, *p* = 0.001), from 47.93 mL to 47.15 mL in ASY (Δ = −0.78 mL, *p* = 0.001), and from 44.87 mL to 43.81 mL in SEV (Δ = −1.06 mL, *p* = 0.001). The left parietal lobe decreased similarly across groups. ICV-normalized differences reached significance only in CTL and ASY, but not in SEV. This finding contrasts with the baseline MRI, in which the parietal lobes-especially in SEV-showed marked cross-sectional atrophy.

**Occipital lobes:** Occipital volumes remained relatively stable. For instance, in the left occipital lobe, SEV showed a small decrease from 34.61 mL to 34.20 mL (Δ = −0.41 mL, *p* = n.s.). No group showed significant longitudinal change in normalized occipital volumes. This finding confirms the result of the baseline MRI, indicating that the occipital lobes were largely spared from COVID-19-related volumetric effects.

In summary, while the initial MRI identified early cortical changes in the frontal, parietal, and thalamic regions, these were not confirmed as progressive in the 12-month follow-up. Instead, the right hippocampus, previously unremarkable, showed significant, severity-dependent atrophy over time. These longitudinal findings show a severity-dependent reduction in right hippocampal volume, with no evidence of widespread progressive atrophy elsewhere.

**Figure 2:**
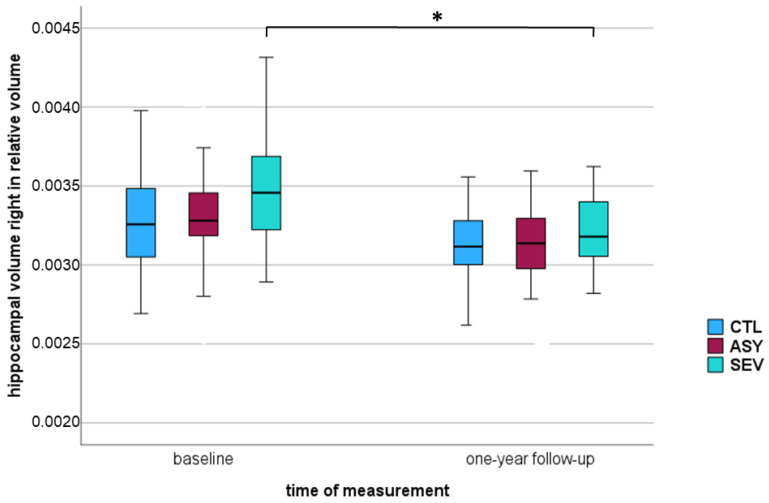
Boxplot of relative right hippocampal volume in three groups—healthy controls (CTL), asymptomatic COVID-19-recovered (ASY), and severe COVID-19-recovered (SEV): at baseline and one-year follow-up. The vertical axis represents hippocampal volume normalized to intracranial volume (ICV). Boxes represent the interquartile range (IQR), horizontal lines within boxes indicate the median, and whiskers represent minimum and maximum values within 1.5 × IQR. * *p* < 0.005 considered statistically significant (marked with *).

Comparing all COVID-19-recovered participants (ASY + SEV) with healthy controls, smaller volumes were observed in the right hippocampus of COVID-19-recovered participants at the 12-month follow-up. The relative changes were CTL: 0.00% ∆, ASY: −3.03% ∆, and SEV: −8.57% ∆ (all values indicate percentage change, representing the mean difference between baseline and follow-up in each group). A significant reduction over time, consistent across all groups was detected (*p* = 0.001). At the 12-month follow-up, we observed a significant decline in right-hippocampal volume within the SEV group (*p*^#^ = 0.001 *), paralleled by a significant reduction in ASY (*p*^#^ = 0.001 *) and a change that did not reach statistical significance in CTL (*p*^#^ = 0.005 †). Stars in Table 3 and Table 4 indicate these within-group changes as assessed by Wilcoxon tests (*p*^#^). The rightmost column of the tables reports the time × group interaction from repeated-measures ANOVA, showing whether the amount of change differed across groups. Here, the effect did not reach statistical significance (*p* = 0.027), although the pattern was consistent with a steeper decline in SEV compared with CTL and ASY. These results suggest a time-dependent decrease in brain volume, with the trajectory differing between groups and the most pronounced decline in the severe COVID group. Across all three groups, including the healthy controls, only a minimal overall volume reduction was observed over one year, remaining within the measurement variance. Nonetheless, significant differences were evident between asymptomatic and severe COVID-19-recovered participants. Figure 2 shows a statistically significant decrease in the percentile of the right hippocampus observed among those recovered from severe COVID-19.

## 4. Discussion

The hippocampus plays a central role in episodic memory, spatial navigation, with strong functional lateralization: the left side mainly supporting verbal episodic functions and the right side supporting visuospatial memory [25,26]. Prior structural and functional imaging work has repeatedly shown that right hippocampal atrophy is associated with deficits in navigation, spatial processing, and scene recognition [25,26,27,28,29,30]. This functional profile suggests that the right hippocampus may be selectively susceptible to systemic inflammatory and metabolic stressors relevant to viral infections. High-resolution MRI and neuropathological evidence indicate that specific hippocampal subregions, most prominently CA1 and CA3, exhibit heightened vulnerability to metabolic disturbance, inflammatory activation, and microvascular stress. Such susceptibility has been documented in conditions characterized by systemic or post-infectious inflammation and aligns with mechanisms proposed in recent experimental and clinical work [31,32]. Such mechanisms, neuroinflammation, hypoxia, and microvascular dysregulation, have been implicated in post-COVID neurological sequelae [31,32]. These considerations provide the anatomical and functional context for interpreting the selective right hippocampal changes observed in our longitudinal cohort. Recent high-resolution MRI and network analysis studies demonstrate that the hippocampus operates within a broader medial temporal prefrontal parietal network architecture [33]. Connectivity between the right hippocampus and prefrontal as well as parietal regions is critically involved in supporting visuospatial navigation, shifts in attentional focus, and the integration of memory-related information [34]. Disruption of these large-scale networks, particularly within the default-mode and medial temporal memory systems, has been reported in viral and inflammatory syndromes and may contribute mechanistically to the right hippocampal atrophy observed in our severe COVID-19 subgroup [15,22,23,34].

This functional profile is consistent with the memory and attention complaints frequently reported in long COVID cohorts. Although direct cognitive volumetric correlations are beyond the scope of this analysis, the right hippocampal vulnerability observed here aligns with domains commonly affected in post-COVID patients, particularly episodic memory and visuospatial attentional functions.

Using MRI follow-up with quantitative AI-based volumetry, we identified long-term effects of COVID-19 on brain volume. Baseline scans showed grey matter loss and focal atrophy in COVID-19-recovered participants, particularly in the frontal and parietal lobes and the right thalamus. The frontal and parietal lobes chiefly underpin executive and attentional control, visuospatial processing, and higher-order integration, while the right thalamus modulates these cortical systems, supporting alertness, working memory, and attention switching [14,15].

To contextualize the longitudinal effect size, right hippocampal volume decreased from 4.21 ± 0.44 to 3.89 ± 0.31 mL in SEV (Δ = −0.32 mL; ≈−7.6%; Wilcoxon *p*^#^ = 0.001), with smaller declines in ASY (Δ = −0.24 mL; ≈−5.6%; *p*^#^ = 0.001) and CTL (Δ = −0.17 mL; ≈−4.0%; *p*^#^ = 0.001). ICV-normalized changes paralleled these findings (SEV Δ = −0.0003; ASY Δ = −0.0001; CTL minimal), indicating a severity-dependent gradient over 12 months.

Previous large-scale longitudinal work, most notably the UK Biobank analysis by Douaud et al., has already demonstrated COVID-19-related grey matter loss in olfactory and limbic cortices, including hippocampal regions [15]. The key advance of our study is not to reconfirm this association, but to characterize the subsequent trajectory of these changes by repeatedly scanning a clinically well-characterized cohort of asymptomatic/mild and severe COVID-19 cases and matched controls over a full 12-month interval using quantitative AI-based volumetry. In this setting, we observe that early fronto-parietal and temporal volume reductions remain largely stable, whereas right hippocampal atrophy continues to evolve and exhibits a clear severity-dependent gradient, thereby refining the temporal and regional specificity of COVID-19-associated hippocampal changes.

Alternative explanations merit consideration. Slightly larger relative right hippocampal values at baseline in SEV likely reflect case-mix (e.g., age, comorbidities, head size), normal segmentation variability, or chance findings from multiple ROI comparisons rather than true structural enlargement. Although regression to the mean is a theoretical concern when baseline values are elevated, SEV baselines were only marginally higher, and the longitudinal decline was supported by within-group tests (SEV *p*^#^ = 0.001) and a time × group trend in RM-ANOVA, arguing against regression as the sole explanation.

Methodological factors also warrant caution. ICV normalization can amplify small errors if ICV is underestimated; automated AI-based segmentation may slightly overestimate small structures under favourable contrast; and multiple regional comparisons increase the risk of spurious findings, which we mitigated via a conservative significance threshold (*p* < 0.005). Assumptions of repeated-measures ANOVA are unlikely to be violated with two time points but should still be acknowledged. Taken together, these considerations do not readily account for the selective, severity-dependent right hippocampal atrophy we observed.

In line with previous studies [35,36,37], our longitudinal analysis revealed no evidence of generalized volume gain in a 12-month period or widespread progression, except for the right hippocampus [35,36,37]. Díez-Cirarda et al. similarly reported hippocampal volume loss, microstructural alterations, and hypoperfusion, correlated with deficits in attention and memory [22], and further work from this same group suggests that hippocampal damage and ongoing neuroinflammation may contribute to cognitive deficits in post-COVID patients [38]. Likewise, Zorzo et al. showed hippocampal volume reductions, altered connectivity, and hypometabolism in COVID-19 survivors in MRI and PET studies [23]. Our selective right hippocampal atrophy without progressive cortical loss elsewhere aligns with a model in which hippocampal injury and possibly altered hippocampo-cortical coupling contribute to attentional inefficiencies after COVID-19, particularly in more severe cases.

Future research should incorporate multi-modal imaging to further characterize microstructural and neuroinflammatory alterations underlying the observed volumetric changes. Subfield-specific hippocampal analyses, longitudinal cognitive testing, and extended follow-up periods (24–36 months) will be essential for determining whether hippocampal atrophy persists, stabilizes, or progresses over time. Integrating imaging data with immunological and clinical biomarkers may also help identify mechanistic pathways and risk factors for long-term neurological sequelae after COVID-19.

Many individuals report persistent fatigue and cognitive impairment after acute COVID-19, driving research into their neurobiological basis and possible treatments [39]. Serrano del Pueblo et al. reported cortical thinning in the left posterior superior temporal gyrus, including Wernicke-associated speech areas, together with widespread white matter abnormalities, particularly in frontal and temporal regions [40]. Reduced white-matter integrity correlated with cognitive deficits, such as poor episodic memory, verbal fluency, and attention, suggesting structural changes underlying these impairments. In that study, the MRI scans were conducted approximately 15–16 months after acute COVID-19 infection, providing a long-term view of post-COVID effects compared with controls who recovered from COVID-19 without lasting symptoms [38]. In contrast, our data revealed significant volume loss only in the right hippocampus relative to controls. Serrano Del Pueblo et al. did not explicitly stratify their 83 long-COVID patients by the severity of their initial COVID-19 infection (mild or severe), which might explain the observed disparity in brain volume differences between the different study groups, in addition to different volumetric measurement approaches.

Recently, Mohammadi et al. reported reduced grey matter volume and cortical thickness in cortical and subcortical regions, particularly in the frontal, temporal, parietal lobes, and cerebellum [41]. In that systematic review, a total of 1219 participants with post-COVID conditions across 25 studies were included, with the MRI scans in the analyzed studies ranging from several weeks to two years post-COVID-19 infection. The findings highlight widespread structural, functional, and perfusion-related brain changes in post-COVID patients, underscoring the need for longitudinal studies to track progression and link these changes to symptoms. Fineschi et al. observed no significant macrostructural or microstructural differences but found altered functional connectivity in the right middle frontal gyrus, associated with attention processes [42].

Despite early brain volume reductions shortly after infection, our current data indicate that this decline does not continue over the first year post-infection, except in the right hippocampus. This apparent stabilization suggests that the initial loss may represent a largely fixed change related to the acute phase of the illness or its immediate aftermath.

Reports on post-COVID cognition remain heterogeneous: some studies demonstrate worse performance compared with controls, others report no differences among recovered individuals, and some show selective domain impairments [39,43,44]. In our cohort, early volumetric reductions after infection did not generally progress over the following year, except in the right hippocampus, suggesting relative structural stabilization elsewhere.

Future research should incorporate multimodal imaging approaches, including diffusion tensor imaging, functional MRI, and PET, to further characterize the microstructural and neuroinflammatory alterations underlying the observed volumetric changes. Subfield-specific hippocampal analyses, longitudinal cognitive testing, and extended follow-up periods (24–36 months) will be essential to determine whether hippocampal atrophy persists, stabilizes, or progresses over time. Integrating imaging data with immunological and clinical biomarkers may help identify mechanistic pathways and risk factors for long-term neurological sequelae after COVID-19. Future work should also assess whether early volumetric changes, particularly right-hippocampal reductions, correlate with longitudinal trajectories of memory and attention, and whether partial recovery occurs. Larger, multi-centre cohorts with harmonized imaging protocols and longer follow-up are needed to verify the persistence or resolution of these changes beyond one year.

Access to pre-infection imaging such as in UK Biobank paradigms would help establish directionality and pre-morbid baselines for hippocampal measures and other regions [15].

In conclusion, our longitudinal data suggest that most early cortical and thalamic changes after COVID-19 remain stable over the first post-infection year, whereas the right hippocampus shows a delayed, severity-dependent decline. To our knowledge, this is the first single-centre longitudinal study to apply AI-assisted quantitative volumetry to track hippocampal and whole-brain volumes over a full year across both asymptomatic/mild and severe cases. These findings extend prior cross-sectional or shorter-term observations [15,22,23,38] by delineating a selective right-hippocampal trajectory.

Limitations include the single-centre design; an analyzed sample of 112 participants (below the a priori target of 126); loss to follow-up; absence of pre-infection imaging; and reliance on automated segmentation and ICV normalization, which may introduce small measurement errors despite quality control. Multiple regional comparisons raise the possibility of false-positive findings; we mitigated this via a conservative significance threshold, but residual risk remains.

Moreover, the present study did not include structure-function analyses or correlations with longitudinal cognitive measures, which limits the ability to directly relate volumetric changes to clinical symptom trajectories. Future multimodal studies combining serial neuroimaging with standardized cognitive follow-up will be essential to clarify these structure-function relationships.

Finally, although some volumetric changes reached statistical significance, their absolute magnitude was small, and the clinical relevance of such subtle structural differences at the individual level remains uncertain. Larger multicentre studies with extended follow-up and harmonized cognitive assessments will be required to determine whether these small effects translate into meaningful functional consequences.

Given the exploratory nature of our analyses and the large number of regions examined, findings with *p*-values between 0.005 and 0.05 should be interpreted as uncorrected, descriptive and hypothesis-generating rather than statistically conclusive. As outlined in the Methods, we deliberately applied a conservative threshold of *p* < 0.005 to reduce the risk of false-positive results; uncorrected findings trend-level effects must therefore be viewed with appropriate caution.

Collectively, our results motivate extended follow-up in larger, multi-centre cohorts, ideally with pre-infection baselines and harmonized cognitive batteries, to clarify the durability and clinical relevance of selective hippocampal change after COVID-19.

## Figures and Tables

**Table 1:** Characteristics of study participants (CTL, ASY, SEV).

Characteristics	Measurement ^b^	Healthy Control Subjects (CTL)	Asymptomatic (ASY)	Severe COVID-19 (SEV)	Total	*p*-Value ^a^
*n*		38	36	38	112	
Age (years)	1	47.0 ± 13.3	45.7 ± 12.4	51.6 ± 11.8	47.7 ± 12.7	0.612
	2	49.2 ± 13.0	47.7 ± 12.2	51.6 ± 11.8	49.5 ± 12.4	0.406
Gender (m:f)		23:15	20:16	20:18	63:49	0.492
Height (cm)		175.1 ± 10.9	173.6 ± 10.4	172.4 ± 8.8	173.7 ± 10.1	0.167
Weight (kg)		81.6 ± 17.1	84.3 ± 25.6	82.3 ± 18.4	82.3 ± 20.5	0.871
BMI		26.3 ± 4.7	28.2 ± 10.4	27.3 ± 5.6	27.2 ± 7.2	0.562

^a^ Calculated with Kruskal–Wallis test; statistical significance set at *p* < 0.05. ^b^ Age is given separately for baseline (1) and 12-month follow-up (2). Other characteristics (gender, height, weight, BMI) were recorded at baseline only. CTL: healthy control subjects, ASY: participants with asymptomatic COVID-19, SEV: participants with severe COVID-19 courses.

**Table 2:** Sequence parameters.

Sequence	Pulse Type	Orientation	TR (ms)	TE (ms)	Reconstructed Voxel Size (mm)	Matrix (mm)	Slices
T2w	Turbo spin echo	axial	13.257	90	0.94 × 0.94 × 1	240 × 174	140
SWI	3D fast field echo	axial	31	0	0.6 × 0.6 × 2	384 × 316	145
DWI	*b* values (0, 500, 1000 s/mm^2^)	axial	2725	41	1 × 1 × 5	128 × 127	24
T1w	MPRAGE	sagittal	7.3	3.9	1 × 1 × 1	256 × 256	180

Abbreviations: DWI: diffusion-weighted imaging; MPRAGE: magnetization-prepared-rapid gradient echo; SWI: susceptibility-weighted imaging; T1w: T1-weighted, T2w: T2-weighted; TE: echo time; TR: repetition time.

**Table 3:** Results of volumetric MRI analysis of brain areas and ventricular volumes in CTL, ASY and SEV, given in absolute volumes [mL] at baseline (1) and at 12-month follow-up (2).

Brain Region	Visit	CTL				ASY				SEV				
		Mean	± SD	% Δ	*p* ^#^	Mean	± SD	% Δ	*p* ^#^	Mean	± SD	% Δ	*p* ^#^	*p*-Value
ICV	1	1296.19	127.29	−0.82	0.001	1304.96	143.00	−0.87	0.001	1218.19	121.17	−0.73	0.001	
	2	1285.59	126.76			1293.59	142.24			1209.31	126.71			
Whole brain	1	1272.88	125.22	−1.04	0.001	1284.63	139.87	−1.16	0.001	1199.03	119.73	−1.75	0.001	0.001
	2	1259.60	124.40			1269.70	138.35			1182.38	121.83			0.628
Whole brain white substance	1	561.95	62.67	−0.58	0.010	566.85	74.17	−2.87	0.023	531.94	59.75	−0.90	0.001	0.069
	2	558.68	62.02			550.57	111.02			527.15	58.38			0.431
Whole brain grey substance	1	710.82	69.84	−1.39	0.001	718.39	73.69	−1.83	0.001	663.10	75.30	−1.08	0.001	0.001
	2	700.92	67.99			705.24	72.35			655.93	66.09			0.484
Supratentorial gross cerebral cortex	1	485.19	49.90	−0.46	0.004	483.77	55.54	0.20	0.850	452.33	51.41	−0.51	0.045	0.081
	2	482.94	49.91			484.74	54.40			450.03	51.87			0.086
Frontal right	1	91.38	10.92	−2.06	0.001	91.24	10.36	−1.48	0.001	84.63	9.76	−1.60	0.001	0.001
	2	89.50	10.64			89.89	10.48			83.28	9.71			0.438
Frontal left	1	87.66	10.17	−2.06	0.001	86.19	14.04	1.07	0.025	81.44	9.52	−0.98	0.004	0.498
	2	86.43	10.16			87.11	10.20			80.64	9.43			0.239
Parietal right	1	48.11	5.44	−1.79	0.001	47.93	5.37	−1.63	0.001	44.87	5.76	−2.36	0.001	0.001
	2	47.25	5.38			47.15	5.39			43.81	5.62			0.554
Parietal left	1	50.26	5.63	−1.23	0.001	50.01	5.87	−1.40	0.001	46.72	6.01	−1.41	0.004	0.001
	2	49.64	5.20			49.31	5.70			46.06	5.79			0.954
Precuneus right	1	11.46	1.50	−0.44	0.820	11.48	1.54	0.44	0.214	10.74	1.41	−0.09	0.975	0.931
	2	11.41	1.69			11.53	1.55			10.75	1.49			0.491
Precuneus left	1	12.14	1.40	−1.73	0.259	12.00	1.69	−0.25	0.458	11.18	1.79	−0.09	0.762	0.257
	2	11.93	2.00			11.97	1.71			11.19	1.73			0.345
Occipital right	1	31.96	3.30	5.54	0.001	33.68	10.89	−0.92	0.001	30.07	5.99	3.96	0.001	0.131
	2	33.73	3.64			33.37	4.70			31.26	4.49			0.328
Occipital left	1	38.79	9.38	−3.22	0.338	37.36	7.95	−0.91	0.007	34.29	4.83	2.16	0.001	0.645
	2	37.54	4.23			37.02	5.35			35.03	4.83			0.422
Temporal right	1	71.81	7.56	−0.99	0.001	72.87	8.84	−1.32	0.001	68.05	8.01	−1.31	0.001	0.001
	2	71.10	7.37			71.91	8.69			67.16	7.28			0.806
Temporal left	1	66.43	9.21	1.97	0.026	67.41	10.25	2.34	0.074	63.54	7.03	−2.64	0.706	0.593
	2	67.74	7.34			68.99	7.83			61.86	12.18			0.149
Hippocampus right	1	4.21	0.45	−4.04	0.001 *	4.30	0.47	−5.58	0.001 *	4.21	0.44	−7.60	0.001 *	0.001 *
	2	4.04	0.38			4.06	0.37			3.89	0.31			0.095
Hippocampus left	1	4.02	0.62	−10.20	0.001	5.02	4.49	−28.09	0.001	4.14	0.58	−16.18	0.001	0.001
	2	3.61	0.32			3.61	0.33			3.47	0.29			0.222
Gyrus parahippocampalis right	1	3.28	0.41	−0.30	0.501	3.37	0.44	0	0.279	3.19	0.36	−0.31	0.706	0.541
	2	3.27	0.40			3.37	0.49			3.18	0.34			0.945
Gyrus parahippocampalis left	1	3.43	0.34	−0.29	0.437	3.53	0.37	0.28	0.348	3.21	0.63	3.12	0.708	0.326
	2	3.42	0.38			3.54	0.36			3.31	0.38			0.395
Regio entorhinalis right	1	2.56	0.33	0	0.989	2.59	0.33	0	0.531	2.48	0.26	0	0.832	0.939
	2	2.56	0.32			2.59	0.37			2.48	0.26			0.965
Regio entorhinalis left	1	2.50	0.29	0.40	0.614	2.50	0.34	2	0.291	2.41	0.33	1.24	0.065	0.031
	2	2.51	0.33			2.55	0.39			2.44	0.34			0.463
Nucleus caudatus right	1	3.28	0.47	−0.30	0.375	3.44	0.43	−0.58	0.819	3.18	0.42	−0.63	0.139	0.111
	2	3.27	0.47			3.42	0.46			3.16	0.39			0.946
Nucleus caudatus left	1	2.89	0.41	−0.35	0.127	3.11	0.37	−0.64	0.108	2.83	0.37	0	0.060	0.066
	2	2.88	0.41			3.09	0.37			2.83	0.34			0.527
Putamen right	1	4.18	0.51	0	0.753	4.27	0.45	−0.23	0.985	4.00	0.46	−1.50	0.085	0.090
	2	4.18	0.50			4.26	0.44			3.94	0.46			0.084
Putamen left	1	4.27	0.52	0	0.957	4.38	0.49	−0.23	0.497	4.09	0.45	−2.20	0.075	0.085
	2	4.27	0.50			4.37	0.48			4.00	0.63			0.211
Pallidum right	1	1.44	0.18	−0.69	0.248	1.45	0.15	−0.69	0.405	1.37	0.15	1.46	10.000	0.979
	2	1.43	0.18			1.44	0.17			1.39	0.14			0.065
Pallidum left	1	1.38	0.17	0.72	0.439	1.43	0.17	−1.40	0.394	1.33	0.15	−1.50	0.182	0.177
	2	1.39	0.17			1.41	0.16			1.31	0.15			0.145
Thalamus right	1	8.16	0.82	0	0.975	8.30	0.83	−0.12	0.526	7.63	0.81	−0.26	0.509	0.505
	2	8.16	0.81			8.29	0.79			7.61	0.82			0.850
Thalamus left	1	8.48	0.88	−0.12	0.682	8.39	1.53	2.98	0.716	7.94	0.81	0	0.946	0.245
	2	8.47	0.89			8.64	0.91			7.94	0.82			0.223
Brainstem	1	27.26	3.92	−1.06	0.002	26.39	2.99	4.28	0.001	24.58	2.58	4.07	0.001	0.064
	2	26.97	4.39			27.52	2.95			25.58	2.37			0.147
Mesencephalon	1	7.43	0.93	11.31	0.001	7.28	0.93	7.55	0.001	6.75	0.84	8.00	0.001	0.001
	2	8.27	3.03			7.83	0.82			7.29	0.63			0.735
Pons	1	14.66	1.88	1.23	0.001	14.36	1.73	2.65	0.001	13.42	1.44	2.16	0.001	0.001
	2	14.84	1.80			14.74	1.68			13.71	1.43			0.044
Cerebellar grey matter	1	110.33	11.24	−0.87	0.006	112.49	10.11	−1.64	0.001	103.58	9.64	−1.70	0.001	0.001
	2	109.37	11.21			110.65	9.62			101.82	8.96			0.127
Left ventricle	1	10.91	7.50	16.22	0.001	8.78	5.35	27.90	0.001	8.37	4.60	35.84	0.001	0.001
	2	12.68	7.41			11.23	6.89			11.37	7.50			0.288
Right ventricle	1	10.52	6.27	5.89	0.001	9.54	5.18	9.85	0.043	8.98	5.34	14.70	0.001	0.001
	2	11.14	6.38			10.48	6.22			10.30	6.84			0.513
Third ventricle	1	0.71	0.41	19.72	0.001	0.79	0.37	−3.80	0.692	0.76	0.36	65.79	0.001	0.077
	2	0.85	0.36			0.76	0.33			1.26	2.05			0.176
Fourth ventricle	1	1.17	0.35	12.82	0.001	1.23	0.40	14.63	0.001	1.06	0.36	278.30	0.001	0.239
	2	1.32	0.36			1.41	0.37			4.01	1.67			0.365

ICV: intracranial volume; CTL: healthy control subjects, ASY: participants with asymptomatic COVID-19, SEV: participants with severe COVID-19 courses. All volumes were measured at two visits: (1) baseline MRI and (2) 12-month follow-up MRI. Values are presented as means ± SD; Δ = absolute change between visits; ^#^ *p*-value of the Wilcoxon test for the change between visits within each group. The rightmost *p*-value column reports repeated measures ANOVA the effect of visit (within-subjects factor) across the three groups (between-subjects factor). * *p* < 0.005 considered statistically significant (marked with *); Rightmost *p*-value = repeated-measures ANOVA time × group effect (between-group comparison of change). % ∆ percentage difference in the mean values of CTL, ASY and SEV.

**Table 4:** Results of volumetric MRI analysis in CTL, ASY and SEV participants in relative volume normalized to the total intracranial volume (ICV) of each subgroup at baseline (1) and at 12 months (2).

Brain Region	Visit	CTL				ASY				SEV				
		Mean	± SD	% Δ	*p* ^#^	Mean	± SD	% Δ	*p* ^#^	Mean	± SD	% Δ	*p* ^#^	*p*-Value
ICV	1	1.0				1.0				1.0				
	2	1.0				1.0				1.0				
Whole brain	1	0.9820	0.0103	−0.21	0.001	0.9844	0.0074	−0.27	0.001	0.9843	0.0076	−0.63	0.001	0.001
	2	0.9799	0.0103			0.9817	0.0091			0.9781	0.0180			0.148
Whole brain white substance	1	0.4335	0.0161	0.21	0.226	0.4344	0.0172	−0.02	0.078	0.4367	0.0163	−0.21	0.617	0.427
	2	0.4344	0.0148			0.4246	0.0669			0.4358	0.0141			0.466
Whole brain grey substance	1	0.5484	0.0175	−1.53	0.004	0.5505	0.0195	−0.46	0.001	0.5443	0.0305	−0.24	0.001	0.056
	2	0.5455	0.0148			0.5459	0.0176			0.5430	0.0196			0.634
Supratentorial gross cerebral cortex	1	0.3743	0.0129	0.37	0.119	0.3707	0.0174	1.13	0.003	0.3713	0.0156	0.22	0.053	0.003
	2	0.3757	0.0126			0.3749	0.0147			0.3721	0.0173			0.165
Frontal right	1	0.0705	0.0043	−1.28	0.004	0.0699	0.0040	−0.57	0.002	0.0695	0.0039	−0.86	0.064	0.001
	2	0.0696	0.0037			0.0695	0.0037			0.0689	0.0040			0.543
Frontal left	1	0.0676	0.0038	−0.59	0.025	0.0660	0.0083	2.12	0.660	0.0669	0.0037	−0.30	0.983	0.552
	2	0.0672	0.0038			0.0674	0.0038			0.0667	0.0037			0.226
Total frontal volume	1	0.1381	0.0078	−0.94	0.005	0.1360	0.0105	0.66	0.232	0.1363	0.0074	−1.32	0.001	0.111
	2	0.1368	0.0074			0.1369	0.0074			0.1345	0.0070			0.058
Parietal right	1	0.0371	0.0019	−1.08	0.001	0.0367	0.0023	−0.54	0.012	0.0368	0.0022	−1.63	0.004	0.001
	2	0.0367	0.0019			0.0365	0.0023			0.0362	0.0022			0.281
Parietal left	1	0.0388	0.0022	−0.52	0.388	0.0383	0.0024	−0.26	0.172	0.0384	0.0020	−0.78	0.321	0.035
	2	0.0386	0.0019			0.0382	0.0024			0.0381	0.0021			0.944
Total parietal volume	1	0.0759	0.0039	−0.66	0.005	0.0751	0.0046	−0.53	0.044	0.0752	0.0041	−1.20	0.013	0.001
	2	0.0754	0.0036			0.0747	0.0045			0.0743	0.0041			0.562
Precuneus right	1	0.0088	0.0008	1.14	0.035	0.0088	0.0007	1.14	0.001	0.0088	0.0007	1.14	0.010	0.019
	2	0.0089	0.0009			0.0089	0.0007			0.0089	0.0007			0.469
Precuneus left	1	0.0094	0.0008	−1.06	0.133	0.0092	0.0007	−3.26	0.099	0.0092	0.0009	0	0.062	0.808
	2	0.0093	0.0013			0.0089	0.0007			0.0092	0.0008			0.364
Total precuneus volume	1	0.0182	0.0014	−0.55	0.042	0.0180	0.0013	1.11	0.004	0.0180	0.0015	0	0.496	0.672
	2	0.0181	0.0022			0.0182	0.0014			0.0180	0.0015			0.457
Occipital right	1	0.0247	0.0019	6.48	0.001	0.0258	0.0074	0	0.001	0.0247	0.0056	4.45	0.001	0.066
	2	0.0263	0.0018			0.0258	0.0020			0.0258	0.0021			0.431
Occipital left	1	0.0299	0.0082	−2.34	0.071	0.0286	0.0044	0	0.001	0.0281	0.0023	2.85	0.001	0.998
	2	0.0292	0.0022			0.0286	0.0022			0.0289	0.0023			0.389
Total occipital volume	1	0.0546	0.0084	1.65	0.001	0.0547	0.0109	−0.73	0.001	0.0528	0.0064	3.79	0.001	0.253
	2	0.0555	0.0037			0.0543	0.0039			0.0548	0.0042			0.636
Temporal right	1	0.0554	0.0026	−0.18	0.464	0.0558	0.0029	−0.36	0.172	0.0559	0.0033	−0.54	0.141	0.212
	2	0.0553	0.0026			0.0556	0.0029			0.0556	0.0031			0.874
Temporal left	1	0.0513	0.0053	2.73	0.001	0.0517	0.0060	3.29	0.001	0.0522	0.0030	−1.92	0.021	0.238
	2	0.0527	0.0025			0.0534	0.0026			0.0512	0.0087			0.140
Total temporal volume	1	0.1067	0.0067	1.22	0.007	0.1075	0.0081	1.40	0.051	0.1080	0.0058	−1.02	0.519	0.407
	2	0.1080	0.0048			0.1090	0.0052			0.1069	0.0101			0.144
Hippocampus right	1	0.0032	0.0003	0	0.005 †	0.0033	0.0003	−3.03	0.001 *	0.0035	0.0004	−8.57	0.001 *	0.001 *
	2	0.0032	0.0002			0.0032	0.0003			0.0032	0.0002			0.027 †
Hippocampus left	1	0.0031	0.0004	−9.68	0.001	0.0038	0.0031	−26.32	0.001	0.0034	0.0005	−14.71	0.001	0.001
	2	0.0028	0.0002			0.0028	0.0003			0.0029	0.0002			0.217
Total hippocampus volume	1	0.0063	0.0007	−4.76	0.001	0.0071	0.0031	−15.49	0.001	0.0069	0.0008	−11.59	0.001	0.001
	2	0.0060	0.0004			0.0060	0.0005			0.0061	0.0004			0.210
Gyrus parahippocampalis right	1	0.0025	0.0002	0	0.261	0.0026	0.0002	0	0.006	0.0026	0.0002	0	0.157	0.200
	2	0.0025	0.0002			0.0026	0.0003			0.0026	0.0002			0.960
Gyrus parahippocampalis left	1	0.0026	0.0002	3.85	0.249	0.0027	0.0002	0	0.014	0.0026	0.0002	3.85	0.023	0.002
	2	0.0027	0.0002			0.0027	0.0002			0.0027	0.0002			0.363
Total parahippocampal volume	1	0.0052	0.0004	0	0.116	0.0053	0.0004	1.89	0.006	0.0053	0.0003	1.89	0.051	0.011
	2	0.0052	0.0004			0.0054	0.0004			0.0054	0.0004			0.663
Regio entorhinalis right	1	0.0020	0.0002	0	0.372	0.0020	0.0002	0	0.561	0.0020	0.0002	5	0.321	0.117
	2	0.0020	0.0002			0.0020	0.0002			0.0021	0.0002			0.966
Regio entorhinalis left	1	0.0019	0.0002	5.26	0.199	0.0019	0.0002	5.26	0.099	0.0020	0.0002	0	0.006	0.001
	2	0.0020	0.0002			0.0020	0.0003			0.0020	0.0002			0.421
Total entorhinal volume	1	0.0039	0.0004	0	0.243	0.0039	0.0004	2.56	0.258	0.0040	0.0003	2.5	0.013	0.003
	2	0.0039	0.0003			0.0040	0.0005			0.0041	0.0003			0.635
Nucleus caudatus right	1	0.0025	0.0003	0	0.446	0.0026	0.0003	3.70	0.018	0.0026	0.0003	0	0.300	0.427
	2	0.0025	0.0003			0.0027	0.0003			0.0026	0.0003			0.968
Nucleus caudatus left	1	0.0022	0.0003	0	0.845	0.0024	0.0003	0	0.388	0.0023	0.0003	0	0.365	0.280
	2	0.0022	0.0003			0.0024	0.0003			0.0023	0.0002			0.642
Total Nucleus caudatus volume	1	0.0048	0.0006	0	0.429	0.0050	0.0005	0	0.023	0.0049	0.0005	2.04	0.226	0.304
	2	0.0048	0.0006			0.0050	0.0006			0.0050	0.0005			0.911
Putamen right	1	0.0033	0.0003	0	0.071	0.0033	0.0003	0	0.051	0.0033	0.0003	0	0.189	0.165
	2	0.0033	0.0003			0.0033	0.0003			0.0033	0.0003			0.092
Putamen left	1	0.0033	0.0003	0	0.036	0.0034	0.0003	0	0.354	0.0034	0.0003	−2.94	0.648	0.940
	2	0.0033	0.0003			0.0034	0.0003			0.0033	0.0005			0.237
Total Putamen volume	1	0.0066	0.0006	0	0.015	0.0066	0.0005	1.52	0.068	0.0066	0.0005	0	0.335	0.612
	2	0.0066	0.0005			0.0067	0.0005			0.0066	0.0007			0.086
Pallidum right	1	0.0011	0.0001	0	0.103	0.0011	0.0001	0	0.153	0.0011	0.0001	9.09	0.001	0.054
	2	0.0011	0.0001			0.0011	0.0001			0.0012	0.0001			0.073
Pallidum left	1	0.0011	0.0001	9.09	0.007	0.0011	0.0001	0	0.509	0.0011	0.0001	9.09	0.788	0.711
	2	0.0012	0.0001			0.0011	0.0001			0.0012	0.0001			0.168
Total Pallidum volume	1	0.0022	0.0002	0	0.060	0.0022	0.0001	0	0.900	0.0022	0.0002	0	0.141	0.170
	2	0.0022	0.0002			0.0022	0.0001			0.0022	0.0002			0.838
Thalamus right	1	0.0063	0.0004	1.59	0.003	0.0064	0.0003	0	0.023	0.0063	0.0004	0	0.112	0.001
	2	0.0064	0.0004			0.0064	0.0003			0.0063	0.0004			0.723
Thalamus left	1	0.0065	0.0004	1.54	0.003	0.0064	0.0010	4.69	0.003	0.0065	0.0004	1.54	0.003	0.032
	2	0.0066	0.0004			0.0067	0.0003			0.0066	0.0004			0.214
Total Thalamus volume	1	0.0128	0.0008	1.56	0.001	0.0128	0.0011	2.34	0.003	0.0128	0.0009	0.78	0.008	0.004
	2	0.0130	0.0008			0.0131	0.0006			0.0129	0.0009			0.204
Brainstem	1	0.0210	0.0026	0	0.001	0.0202	0.0014	5.45	0.001	0.0202	0.0017	4.95	0.001	0.014
	2	0.0210	0.0028			0.0213	0.0013			0.0212	0.0017			0.141
Mesencephalon	1	0.0057	0.0005	14.04	0.001	0.0056	0.0005	8.93	0.001	0.0055	0.0005	10.91	0.001	0.001
	2	0.0065	0.0024			0.0061	0.0003			0.0061	0.0004			0.767
Pons	1	0.0113	0.0010	2.65	0.001	0.0110	0.0009	3.64	0.001	0.0110	0.0011	3.64	0.001	0.001
	2	0.0116	0.0009			0.0114	0.0008			0.0114	0.0011			0.122
Cerebellar grey matter	1	0.0851	0.0055	0.12	0.960	0.0862	0.0063	−0.35	0.004	0.0850	0.0076	−0.47	0.037	0.001
	2	0.0852	0.0055			0.0859	0.0055			0.0846	0.0072			0.096
Total ventricle volume	1	0.0180	0.0103	11.67	0.001	0.0156	0.0074	17.31	0.001	0.0157	0.0076	39.49	0.001	0.001
	2	0.0201	0.0103			0.0183	0.0091			0.0219	0.0180			0.148
Left ventricle	1	0.0084	0.0055	16.67	0.001	0.0067	0.0037	28.36	0.001	0.0069	0.0035	36.23	0.001	0.001
	2	0.0098	0.0056			0.0086	0.0049			0.0094	0.0057			0.200
Right ventricle	1	0.0081	0.0047	6.17	0.001	0.0073	0.0038	9.59	0.018	0.0074	0.0040	14.86	0.001	0.001
	2	0.0086	0.0048			0.0080	0.0043			0.0085	0.0051			0.411
Total lateral ventricle volume	1	0.0165	0.0100	12.12	0.001	0.0140	0.0072	18.57	0.001	0.0142	0.0073	26.06	0.001	0.001
	2	0.0185	0.0100			0.0166	0.0088			0.0179	0.0105			0.077
Third ventricle	1	0.0005	0.0003	40	0.001	0.0006	0.0003	0	0.975	0.0006	0.0003	66.67	0.003	0.050
	2	0.0007	0.0003			0.0006	0.0003			0.0010	0.0015			0.139
Fourth ventricle	1	0.0009	0.0002	11.11	0.001	0.0009	0.0003	22.22	0.001	0.0009	0.0003	233.33	0.001	0.223
	2	0.0010	0.0002			0.0011	0.0003			0.0030	0.0119			0.356

ICV: intracranial volume; CTL: healthy control subjects, ASY: participants with asymptomatic COVID-19, SEV: participants with severe COVID-19 courses. Results of volumetric MRI analysis in CTL, ASY and SEV participants, given in relative volumes. All volumes were measured at two visits: (1) baseline MRI and (2) 12-month follow-up MRI. All volumes were normalized to the intracranial volume (ICV) for CTL, ASY and SEV participants. Values are presented as means ± SD; Δ = absolute change between visits; ^#^  *p*-value of the Wilcoxon test for the change between visits within each group. The rightmost *p*-value column reports repeated measures ANOVA the effect of visit (within-subjects factor) across the three groups (between-subjects factor). * *p* < 0.005 considered statistically significant (marked with *); 0.005 ≤ *p* < 0.05 are reported descriptively but are not statistically significant (marked with †). Rightmost *p*-value = repeated-measures ANOVA time × group effect (between-group comparison of change). % ∆ percentage difference in the mean values of CTL, ASY and SEV.

## Data Availability

The original contributions presented in this study are included in the article. Further inquiries can be directed to the corresponding author.

## Abbreviations

The following abbreviations are used in this manuscript:

3D MPRAGE	3D T1-weighted magnetization-prepared rapid acquisition gradient echo
3D FLAIR	3D fluid-attenuated inversion recovery
ASY	asymptomatic/mild course of COVID-19
CTL	healthy controls
DWI	diffusion-weighted imaging
SEV	severe course of COVID-19
SWI	susceptibility-weighted imaging
T2w	T2-weighted imaging
TE	echo time
TR	repetition time
